# Dyadic capability in post-stroke transitional care: a study protocol for instrument development and longitudinal evaluation

**DOI:** 10.3389/frhs.2026.1924597

**Published:** 2026-09-23

**Authors:** Eleonora Lombardi, Gianluca Pucciarelli, Federico Cortese, Vincenzo Damico, Belen Gutierrez-Baena, Rosaria Alvaro, Ercole Vellone, Francesco Petrosino, Davide Bartoli

**Affiliations:** 1 Department of Biomedicine and Prevention, University of Rome Tor Vergata, Roma, Italy; 2 Division of Research Methodology, Department of Nursing, Faculty of Nursing and Midwifery, Wroclaw Medical University, Wroclaw, Poland; 3 Aziende Socio Sanitarie Territoriali di Lecco, Lecco, Italy; 4 ‘Salus Infirmorum' University Nursing Centre, University of Cadiz, Cadiz, Spain; 5 Health Professions Directorate, Local Health Authority Rome 3, Rome, Italy; 6 Department of Wellbeing, Health and Environmental Sustainability (BeSSA Department), Sapienza University of Rome, Rome, Italy

**Keywords:** capability, conceptual framework, dyad, educational program, informal caregiver, stroke, study protocol, transitional care

## Abstract

**Introduction:**

Stroke represents a major burden for both survivors and caregivers. Although caregiver preparedness has been widely studied, it is typically assessed through self-reported measures that capture subjective perceptions rather than real-life adaptive functioning. Conventional training approaches, focused on knowledge and technical skills, often fail to support sustainable long-term care. The concept of capability may offer a more comprehensive framework for evaluating caregiving; however, it remains insufficiently operationalized and empirically validated in stroke populations.

**Aims:**

To develop, operationalize, and empirically evaluate dyadic capability in stroke survivor–caregiver dyads.

**Methods:**

Building on a completed and separately published systematic review and integrated concept analysis, this mixed-methods longitudinal study protocol comprises four linked empirical phases: qualitative exploration, instrument development and validation, longitudinal observational evaluation, and intervention evaluation. The protocol has been prospectively registered on the Open Science Framework (OSF): https://osf.io/sjdzp. Qualitative data will be analysed using conventional content analysis and integrated through triangulation. Psychometric evaluation will include Delphi consensus procedures and factor analyses. Quantitative data will be collected at five timepoints (pre-intervention baseline and 1, 3, 6, and 12 months) and analysed using linear mixed-effects and dyadic (Actor–Partner Interdependence) models.

**Expected results:**

The Dyadic Capability Scale is expected to support the assessment of dyadic capability and the empirical refinement of the proposed conceptual framework for adaptive post-stroke care.

**Conclusions:**

This protocol proposes a theoretically grounded and clinically applicable conceptual framework for assessing dyadic capability in post-stroke care.

## 1 Introduction

Stroke is the second leading cause of death and the third leading cause of disability worldwide (1). In Europe alone, more than 1 million new cases and over 6 million prevalent cases were reported in 2021 (2). The global economic burden of stroke exceeds $890 billion annually (1).

After the acute phase of stroke, many stroke survivors continue to live with long-term disabilities (3). The most common include physical impairments (4), aphasia (5), and hemiplegia (6). These impairments are often accompanied by psychological disorders (7, 8). Such conditions limit the ability of stroke survivors to perform daily activities and participate in social life (9). Once rehabilitation is complete, care is usually provided at home by informal caregivers, such as family members or friends (10).

Caregiving entails a substantial burden, with significant repercussions on psychological, physical (11), and financial wellbeing (12). This negatively affects overall quality of life (13) (12). The first few months after discharge are critical for both stroke survivors and their caregivers (12, 14).

### 1.1 Background

Preparedness refers to the caregiver's perceived readiness to provide care (15) and is usually assessed through self-report instruments reflecting subjective perception (16). In the caregiving literature (17–19), several closely related constructs have been used to describe different dimensions of caregiving adaptation, often with partial conceptual overlap. In the health sciences, capacity generally refers to the objective resources available (e.g., physical health, time, environmental and organizational resources) (20), whereas competence denotes the effective execution of specific caregiving tasks (21). Ability reflects the underlying physical, cognitive, and emotional potential to engage in care-related activities (22). These constructs have been extensively investigated in stroke caregiving; however, they have been almost exclusively conceptualized as individual caregiver attributes, whereas the interdependent, relational, and context-sensitive functioning of the stroke survivor–caregiver dyad has remained largely unexplored. Over time, preparedness may evolve into mastery (23, 24) reflecting the perceived sense of control, confidence, and self-efficacy in managing caregiving demands as experience accumulates over time; however, none of these stages necessarily ensures that care can be effectively enacted under real-life contextual and relational constraints. In contrast, capability, as defined within Sen's Capability Approach (25), does not replace these constructs nor merely reflect perceived competence or control. A caregiver may feel “prepared” (high preparedness) to assist the stroke survivor with transfers and ambulation but may not actually be able to do so safely due to physical barriers in the home, lack of support, or poor cooperation from the survivor. In this case, preparedness is high, but capacity is low, resulting in a greater risk of falls and fatigue. Similarly, on the survivor side, a person may feel fully prepared to manage their medication, yet be unable to do so in practice: a change in dosage, temporary unavailability of the prescribed medication, or the absence of someone to confirm the new schedule can convert high preparedness into low real capability. Here too, the resource (knowledge of the prescribed medication regimen) is present, but the freedom to convert it into safe, autonomous action is constrained by contextual and relational conditions. Key tasks such as medication management (26), patient mobilization, including transfers and ambulation (27), and recognition of warning signs (28) require not only technical competence but also the dyadic ability to adapt, negotiate decisions, and collaborate effectively.

Stroke survivor and caregiver outcomes are deeply interdependent (29), meaning that adaptive functioning depends not only on the caregiver's skills but also on the survivor's engagement, autonomy, communication, and emotional state. Although dyadic educational programs have proven effective in transitional care (30–34), they often emphasize patient needs while overlooking the caregiver's active role and the joint adaptation of the dyad.

The concept of capability extends beyond preparedness by incorporating contextual, relational, and systemic factors that shape how resources are mobilized and how care is enacted (25, 35). Modern healthcare increasingly expects individuals and families to play an active role in navigating services and managing care transitions (36), yet many dyads may lack the conditions needed to effectively fulfil these expectations, leading to unmet needs. For this reason, studying capability offers a more realistic and comprehensive evaluation of the dyadic functioning, particularly in complex and unpredictable home-care environments (37).

Capability is not an attribute of one individual but a shared, co-constructed property of the stroke survivor–caregiver dyad, emerging from their interaction and the context in which care takes place. Accordingly, dyadic capability may represent a more comprehensive construct for understanding post-stroke adaptation at the level of the relationship, rather than the caregiver alone. Moreover, while education and training may enhance related constructs such as preparedness, self-efficacy, and competence, it remains largely unknown whether and to what extent these resources are translated into everyday care practice under real-life conditions.

Several capability-based or capability-relevant instruments have already been developed, including ICECAP-A for adults (38), ICECAP-O for older people (39), ICECAP-SCM for end-of-life care (40), the Carer Experience Scale for informal carers (41), and ASCOT for social care-related quality of life (42). These measures assess capability or broader care-related quality of life at the individual level and have been developed largely for outcome measurement and economic evaluation. However, none was designed to assess capability as a dyadic construct in post-stroke transitional care or to distinguish available capability from observed enactment.

To date, no study has explicitly theorized, operationalized, and empirically measured capability as a dyadic construct grounded in the Capability Approach within the context of stroke care. In addition, no validated instrument currently exists to measure dyadic capability grounded in the Capability Approach within stroke survivor–caregiver populations.

## 2 Theoretical background

This study is guided by a conceptual framework grounded in the Capability Approach, originally developed by Amartya Sen (25) and later expanded by Martha Nussbaum (43), who introduced the notion of “flourishing” to represent the various ways people can reach their full potential. In this perspective, educational programs are viewed as enablers of capability: they are not limited to transmitting technical competence, but aim to support adaptive functioning, autonomous decision-making, and meaningful participation in care settings. This theoretical lens informs the study design, guides the selection of variables, and supports the development of a robust conceptual framework. This approach emphasizes individuals' substantive freedoms, that is, the real opportunities to achieve valued outcomes, even under conditions of vulnerability.

Within Sen's Capability Approach, capabilities refer to the real opportunities or substantive freedoms to achieve functionings that a person has reason to value, whereas functionings refer to the states and activities actually achieved (25, 35). The conversion of resources into capabilities is shaped by conversion factors, commonly distinguished in the capability literature as personal, social, and environmental factors (44). Personal factors include individual characteristics such as physical condition, skills, and knowledge; social factors include social norms, institutional arrangements, and social structures; and environmental factors concern features of the physical and built environment that may facilitate or constrain opportunities (44). The capability set comprises alternative potential functionings from which a person can choose; therefore, choice and agency are relevant to which functionings are ultimately achieved (25).

### 2.1 Conceptual foundation

A systematic review and integrated concept analysis conducted using the Walker and Avant method (45) has been completed and published separately (46). This work constitutes the conceptual foundation of the present protocol and identified the defining attributes, antecedents, consequences, and empirical referents of dyadic capability in stroke survivor–caregiver dyads. Dyadic capability was defined as:

“The freedom and opportunity, activatable and transferable with expert support, to mobilize personal, social, and contextual resources through self-efficacy, resilience, and motivation to adapt and improve continuously in managing care and daily life” (46).

Conceptually, dyadic capability is understood within the broader structure of the Capability Approach, in which available resources are converted into real opportunities through personal, social, and environmental conversion factors, while choice and agency influence whether available capabilities are ultimately enacted as achieved functionings (25). Within the applied dyadic model proposed in this protocol, self-efficacy, resilience, and motivation are retained as capability-related psychological domains derived from the published concept analysis but are not interpreted as Senian conversion factors. Rather, they are hypothesised to influence the exercise of choice and agency and the enactment of available capabilities in everyday care.

### 2.2 Antecedents and factors associated with dyadic capability

Based on the findings of the conceptual foundation and the existing literature, a range of predictive factors has been identified that contribute to the development of capabilities within the stroke survivor–caregiver dyad. These factors have been classified into three main categories: individual, relational, and contextual. Each dimension is discussed in detail in the following sections.

#### 2.2.1 Individual factors

##### 2.2.1.1 Sociodemographic variables

Among sociodemographic variables, gender, education, and age emerge as predictors of capability.

Female caregivers report higher burden and fatigue, often neglecting personal needs due to traditional caregiving roles (47, 48). Conversely, higher education is linked to greater caregiving competence (49–51).

In stroke survivors, older age and functional dependence are strongly associated with disability (52).

##### 2.2.1.2 Caregiver's physical health

The physical health of the caregiver is an important determinant of their ability to provide effective support (47, 53, 54). Poor caregiver health reduces capability (55) and increases strain (54, 56).

##### 2.2.1.3 Stroke survivor's physical health

Greater stroke severity increases caregiver burden and lowers QoL, while higher survivor functioning is linked to better caregiver wellbeing (57). Consistent with Sen's Capability Approach (25), reduced functioning is not equated with the capability outcome itself; rather, the survivor's physical health and functional status are treated in this framework as antecedent conditions that shape care demands and the resources the dyad must mobilise, thereby influencing, but not defining, the development of dyadic capability.

##### 2.2.1.4 Mental health

The caregiver's mental health substantially affects the ability to manage care, especially under stress during role transition (54). High levels of uncertainty are common, with almost half reporting symptoms of depression or anxiety (55, 58). Identifying stress-related factors can help anticipate responses to target interventions (59, 60). In stroke survivors, underdiagnosed depression and anxiety limit autonomy despite neurological recovery (61). These symptoms (61) can seriously compromise an individual's real opportunities to live autonomously and meaningfully, thus limiting their capability regardless of physical improvements.

##### 2.2.1.5 Spirituality

Spirituality moderates depressive symptoms and improves QoL (62). Spiritual factors provide purpose and inner strength, supporting caregivers in coping with emotional and practical challenges (53, 63). Thus, spirituality can be considered both an individual and relational predictor of capability, fostering meaning and resilience under stress.

#### 2.2.2 Relational factors

##### 2.2.2.1 Mutuality

Relational quality can influence the effectiveness of educational interventions (64). Caregivers report greater satisfaction when the relationship is emotionally meaningful—an element that can strengthen motivation in daily care management (8). Within this framework, mutuality emerges as a potential predictor of caregiving capability by enhancing dyadic engagement.

##### 2.2.2.2 Communication

Communication is a key component in managing care within the dyad. Improved communication fosters a deeper understanding of the needs of others, thus supporting the development of caregiving capability (65).

Poor communication undermines relationships and care, while effective communication fosters collaboration (66, 67). Effective communication, therefore, represents a modifiable predictor of capability, fostering emotional wellbeing and collaborative care.

#### 2.2.3 Contextual factors

##### 2.2.3.1 Health literacy

Health literacy enables navigation of care and informed decisions (68, 69). In stroke survivors, higher levels of health literacy are associated with greater self-efficacy and functional independence (70). It therefore supports both understanding of care needs and autonomy, central aspects of capability.

##### 2.2.3.2 Socioeconomic factors

Income has been identified as a predictive factor for both members of the dyad. The literature consistently shows that individuals with lower income levels tend to have lower health capability (47, 49). Financial difficulties increase caregiver burden, particularly when compounded by the additional expenses of care (32, 51, 71). Socioeconomic disadvantage can act as a structural constraint on capability by limiting access to resources and services.

##### 2.2.3.3 Social support and networking

Perceived social support strongly influences caregivers' ability to manage responsibilities and reduces the physical burden of care (47), improves QoL and reduces dropout risk (72). Access to support is shaped by cultural factors, which also affect the adaptability of educational programs (53, 59). Among younger stroke survivors, perceived social support tends to be lower (73). In addition, social support has been shown to mediate the relationship between functional abilities and participation (74).

### 2.3 Theoretical framework

We hypothesize that although dyadic capability is conceptualized at the dyadic level, the real opportunities available to stroke survivors and caregivers may differ in how they are expressed and enacted. For stroke survivors, these opportunities concern adaptation to their condition, participation in shared decision-making, management of residual disabilities, and autonomy in daily life. For caregivers, they concern providing effective support, adapting to evolving needs, managing time and resources, and maintaining emotional resilience.

The bidirectional interaction between caregiver and survivor capability is essential. A competent caregiver can promote the survivor's recovery and independence, while a competent survivor can reduce caregiver burden and facilitate mutual adjustment. Thus, capability is interdependent yet uniquely expressed by each member of the dyad.

At the centre of this framework is the dyadic capability set, representing the real opportunities available to the stroke survivor–caregiver dyad (25, 35). Resources and enabling conditions, including educational interventions, contribute to the capability set, while personal, social, and environmental conversion factors shape the conversion of these resources into real opportunities (44).

Educational programs are recognized as a key to reducing inequalities in capability development (75, 76). By strengthening the resources and enabling conditions available to the dyad, these interventions may support adaptation, autonomy, and the overall QoL of both dyad members (33).

These variables will be operationalized quantitatively in Phase III as predictors and moderators of dyadic capability and related outcomes.

### 2.4 Visual model of the framework

Figure 1 illustrates the proposed conceptual framework of dyadic capability. Distal individual, relational, and contextual factors influence the resources and enabling conditions available to the dyad. The conversion of these resources and enabling conditions into real opportunities is shaped by personal, social, and environmental conversion factors. The resulting dyadic capability set represents the real opportunities available to the stroke survivor–caregiver dyad. Choice and agency influence whether these available capabilities are enacted as achieved functionings.

**Figure 1:**
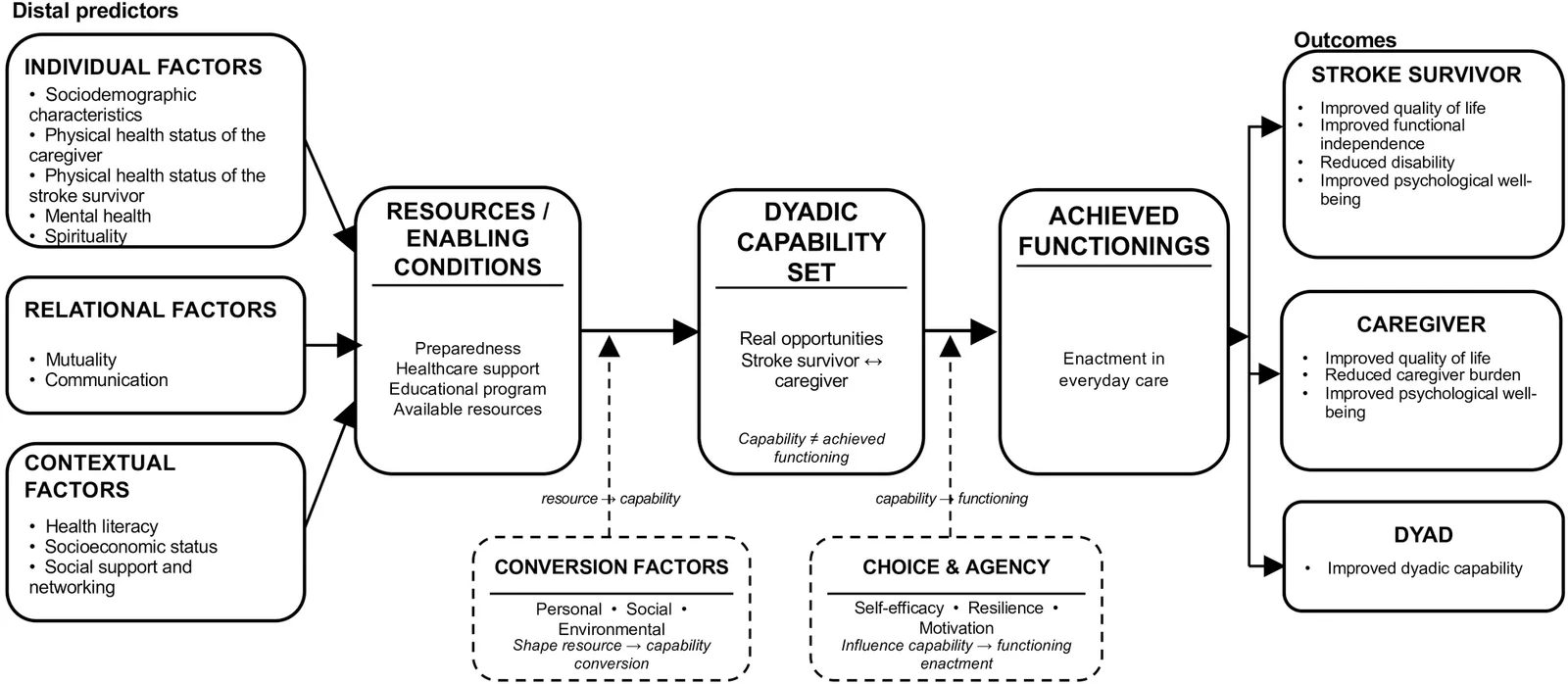
Conceptual framework of dyadic capability. Distal predictors include individual, relational, and contextual factors. These predictors influence the availability of resources and enabling conditions, including preparedness, healthcare support, and educational interventions. The conversion of resources into real dyadic opportunities is shaped by personal, social, and environmental conversion factors. The resulting dyadic capability set represents the real opportunities available to the stroke survivor–caregiver dyad. Choice and agency influence whether these capabilities are enacted as achieved functionings, with self-efficacy, resilience, and motivation hypothesised to influence this process. Achieved functionings may subsequently contribute to outcomes including improved quality of life, improved functional independence, reduced disability, improved psychological well-being, and reduced caregiver burden.

Within the proposed model, self-efficacy, resilience, and motivation are hypothesised to influence this choice and enactment process rather than operate as Senian conversion factors. Achieved functionings may subsequently contribute to outcomes including improved quality of life, improved functional independence, reduced disability, improved psychological wellbeing, and reduced caregiver burden.

## 3 Objectives

### 3.1 Primary objective

To develop, operationalize, and empirically evaluate dyadic capability in stroke survivor–caregiver dyads.

### 3.2 Sub-objectives

1. To explore how capability is perceived, expressed, and experienced by stroke survivors and caregivers through qualitative content analysis of semi-structured interviews.
2. To develop and psychometrically validate a new theory-based instrument, the Dyadic Capability Scale (DCS), for the assessment of capability within stroke survivor–caregiver dyads.

### 3.3 Secondary objectives

1. To examine whether dyadic capability (DCS) predicts key outcomes, including QoL (Stroke Impact Scale 3.0; WHOQOL-BREF), caregiver burden (Caregiver Burden Inventory), and psychological wellbeing (Hospital Anxiety and Depression Scale), through a longitudinal observational study.
2. To examine how sociodemographic, clinical, and psychological resources [Walsh Family Resilience Questionnaire; Revised Caregiving Self-Efficacy Scale; WHOQOL-SRPB; Mutuality Scale; Preparedness for Caregiving Scale; HLS-EU-Q16; Multidimensional Scale of Perceived Social Support (MSPSS)] predict dyadic capability, and whether contextual resources moderate the association between dyadic capability and dyadic outcomes.
3. To evaluate intervention-related changes in dyadic capability (primary endpoint: change in DCS), and in improving QoL (WHOQOL-BREF, SIS 3.0), psychological wellbeing (HADS), functional status (Barthel Index, mRS), and caregiver burden (CBI).

### 3.4 Conceptual hypotheses

Based on this framework, we hypothesize that:

H1. Sociodemographic and clinical characteristics of stroke survivors and caregivers will influence levels of dyadic capability as assessed by the Dyadic Capability Scale (DCS).
H2. The quality of the dyadic relationship, assessed with the Mutuality Scale, will moderate intervention-related changes in dyadic capability over time. H2 is intentionally framed as a focused relational hypothesis testing mutuality as the moderator of intervention responsiveness; other individual factors (preparedness, self-efficacy) are examined as predictors of capability under H3 rather than as moderators here. H2 is designated *a priori* as the primary confirmatory moderation hypothesis of the protocol.
H3. Psychological resources, including resilience (Walsh Family Resilience Questionnaire), spirituality (WHOQOL-SRPB), preparedness (Preparedness for Caregiving Scale), self-efficacy (Revised Caregiving Self-Efficacy Scale), and health literacy (HLS-EU-Q16), will predict higher dyadic capability scores.
H4. Physical health of both members of the dyad, assessed with the Barthel Index and modified Rankin Scale, will facilitate the execution of tasks and the sustainability of capability.
H5. Contextual resources (social support, health literacy, and socioeconomic status) will moderate the association between dyadic capability and dyadic outcomes, strengthening or constraining the translation of capability into improved quality of life and reduced caregiver burden. H5 is designated *a priori* as exploratory and hypothesis-generating.
H6. Higher dyadic capability, as assessed by the Dyadic Capability Scale, will be associated with better outcomes: greater QoL (Stroke Impact Scale 3.0; WHOQOL-BREF), lower caregiver burden (Caregiver Burden Inventory), and reduced psychological distress (Hospital Anxiety and Depression Scale).

## 4 Methods

### 4.1 Study design

This study follows a multi-phase, mixed-methods design aligned with the primary and secondary objectives. Building on a completed and separately published systematic review and integrated concept analysis (46), the protocol comprises four linked empirical phases: qualitative exploration, instrument development and validation, longitudinal observational evaluation, and intervention evaluation.

The evaluation of capability will be embedded within the implementation of a teletraining and telesupport educational program for stroke survivors and caregivers, based on a previously developed study protocol (77). The present study is methodologically informed by the D-STEPS programme but constitutes a distinct research protocol. D-STEPS is used solely as the educational and transitional-care methodology within which dyadic capability will be assessed. The present study addresses a different research question, uses the newly developed Dyadic Capability Scale as its primary outcome, and involves separate prospective data collection. No data from the previously published D-STEPS study will be analysed in the present protocol. The present study will recruit a separate sample of dyads and will not share participants with the previously published D-STEPS study.

Embedding capability assessment in this structured intervention allows us to test the construct in a real-world clinical context, ensuring that its measurement is directly linked to practical outcomes such as caregiver preparedness, QoL, and functional recovery. Reporting follows the guideline appropriate to each component: STROBE for the observational analyses (78), TREND for the single-group pre–post intervention evaluation (79), and COREQ for the qualitative phase (80). A completed SPIRIT checklist is provided as Supplementary Material, with non-applicable items identified according to the single-group, non-randomised design (81).

#### 4.1.1 Conceptual foundation: systematic review and integrated concept analysis

A systematic review and integrated concept analysis was conducted to clarify how the capability construct has been applied in healthcare, rehabilitation, and caregiving, and to identify its defining attributes, antecedents, consequences, and empirical referents within stroke survivor–caregiver dyads. The systematic review followed PRISMA 2020 guidelines (82), and the methodological quality of the included studies was assessed using the JBI critical appraisal checklists (83). The review protocol was prospectively registered in PROSPERO (CRD420250653734). The findings were published separately (46) and provide the conceptual foundation for the empirical phases described in the present protocol. An overview of the study phases, corresponding objectives, designs, and expected outputs is presented in Table 1.

**Table 1:** Study phases with corresponding objectives, designs, and expected outputs.

Phase	Objective addressed	Study design	Expected outputs
Conceptual Foundation	Conceptualize dyadic capability	Systematic review and integrated concept	Defining attributes, antecedents, consequences, and empirical referents of dyadic capability
I. Qualitative study	Explore how capability is perceived, expressed, and experienced	Qualitative content analysis	Empirical grounding and refinement of the proposed operational model of dyadic capability; contribution to middle-range theory development.
II. Instrument Development and Validation	Develop and validate a measurement tool	Mixed-method instrument development; Delphi; psychometric testing	Validated questionnaire for dyadic capability
III. Longitudinal Observational Study	Test the predictive and moderating role of capability	Prospective longitudinal design assessments at baseline (T0, during hospitalisation and before the first programme session) and at 1, 3, 6, and 12 months	Evidence on the predictive value of dyadic capability and the moderating role of contextual resources.
IV. Interventional Study	Evaluate intervention-related changes	Single-group, pre–post quasi-experimental evaluation of the educational/telehealth programme (with no control group)	Evidence on intervention-related changes in dyadic capability and QoL outcomes.

#### 4.1.2 Phase I. Qualitative study

To empirically ground the proposed conceptual framework derived from the published concept analysis, a qualitative study will be conducted using conventional content analysis of semi-structured interviews with stroke survivors and caregivers (84), a methodology previously applied in stroke research (85) to explore post-stroke experiences and caregiving. All interviews will be audio-recorded and manually transcribed verbatim. This phase will explore how capability is perceived and experienced within stroke survivor–caregiver dyads and will inform refinement of the proposed conceptual framework.

Data will be analysed using a systematic, inductive coding process consistent with conventional content analysis (84). To achieve immersion in the data, transcripts will be read repeatedly and supported by analytic summary memos documenting preliminary interpretations (86). Two researchers will independently code an initial subset of transcripts, allowing descriptive codes to emerge directly from the text. Coding discrepancies will be resolved through consensus discussion, in line with established standards for qualitative rigor and credibility (87, 88). Codes will then be grouped into categories based on conceptual similarities and subsequently abstracted into themes through an iterative interpretive process. Interviews will last approximately 45–60 min (range 30–75) and will be conducted online via a secure videoconferencing platform. Potential mode effects related to the online format will be acknowledged and analytically considered.

Interviews will be conducted individually with stroke survivors and caregivers to allow each participant to express their perspective independently and to minimize reciprocal influence during data collection. Data collection will continue until thematic saturation is achieved. All interviews will be conducted by researchers with formal training in qualitative methods and experience in stroke rehabilitation research. Participants for the qualitative phase will be recruited from the same cohort using purposive, maximum-variation sampling, seeking diversity in survivor age and sex, stroke severity, and caregiver relationship and sex. Approximately 20–30 dyads, corresponding to 40–60 individual interviews, are anticipated, with the final sample determined by thematic saturation.

#### 4.1.3 Phase II. Instrument development and validation

Based on findings from the conceptual foundation and Phase I, a measurement instrument will be developed to assess dyadic capability. Content validity will be established through expert panel review and cognitive interviews.

The Delphi process (89) will involve at least two rounds and a multidisciplinary panel of 10–15 experts in stroke care, rehabilitation, and instrument development. Cognitive interviews will be conducted separately with stroke survivors and caregivers to assess item clarity, relevance, and interpretability at the individual level within the dyad (90). Psychometric validation will include exploratory and confirmatory factor analyses, reliability testing (Cronbach's *α*, McDonald's *ω*, test–retest), and construct validity assessment. This phase directly addresses the primary objective of developing and validating a capability measurement instrument. The current candidate item pool was initially derived from the attributes, antecedents, and constructs identified through the conceptual foundation. Before expert review and the Delphi process, a panel of stroke survivors and family caregivers, recruited independently of the study sample, will review the candidate item pool for relevance, comprehensibility, acceptability, and appropriateness of wording. Their feedback will be documented and used to refine the item pool submitted to expert review. Findings from the Phase I qualitative interviews will subsequently be used to refine this pool before expert review, Delphi consensus, cognitive interviews, and item-reduction procedures. The current candidate pool is provisionally organised into four theory-driven domains: self-efficacy, resilience, motivation (capability-related psychological domains), and enactment (applied competence), each articulated across three relational levels: the survivor, the caregiver, and the dyad (couple).

The first three domains (35 self-report items) are intended to be completed independently by each member in role-specific parallel versions, whereas the enactment domain (22 items) is rated by a trained clinical nurse assessor who is not involved in the delivery of the D-STEPS programme, on the basis of competence demonstrated during structured assessments, thereby providing a non-self-reported source of measurement. The instrument is conceptualised as genuinely dyadic on three grounds: it contains couple-level subscales rated at the level of the dyad; dyad-level scores and indices are analysed at the dyadic level; and two dyad-level indices are derived, the Dyadic Discrepancy Index (within-dyad divergence between survivor and caregiver) and the Enactment Gap Index (the difference between self-reported capability-related psychological domains and nurse-observed enactment). We nonetheless acknowledge that the survivor- and caregiver-level subscales are individual self-reports analysed for cross-partner interdependence via the Actor–Partner Interdependence Model, and we distinguish these from the couple-level subscales and indices, which capture emergent dyad-level properties.

#### 4.1.4 Phase III. Longitudinal observational study

To address the secondary objectives, the same cohort of dyads will be followed longitudinally across five assessments aligned to the D-STEPS programme: T0, a single baseline assessment completed during the index hospitalisation and before the dyad receives any component of the programme; T1 (1 month), T2 (3 months), T3 (6 months), and T4 (12 months). These observational analyses will examine whether dyadic capability predicts outcomes such as quality of life and caregiver burden, and whether contextual resources moderate the association between capability and these outcomes. Reporting of the observational analyses follows the STROBE guidelines (78).

#### 4.1.5 Phase IV. Interventional study

To evaluate intervention-related changes associated with the educational programme, a single-group, pre–post quasi-experimental evaluation will be conducted within the same cohort, comparing the baseline assessment (T0, collected during hospitalisation before the first programme session) with the subsequent assessments at T1–T4 to model change over time. Because all dyads receive the D-STEPS programme, no concurrent control group is available; this is acknowledged as a limitation, and causal inference is treated as exploratory. The primary endpoint is the change in dyadic capability (DCS); secondary endpoints are changes in quality of life, psychological wellbeing, functional status, and caregiver burden. The relational quality of the dyad (mutuality) will be tested as the primary, pre-specified moderator of intervention responsiveness (H2). Analyses will use longitudinal mixed-effects and dyadic (APIM) models. Reporting of this interventional component follows the TREND statement for non-randomised evaluations (79). The sequential data analysis plan and the integration of qualitative and quantitative findings across the study phases are illustrated in Figure 2. The overall timeline of the four empirical phases and the assessment sequence within each dyad are presented in Figure 3.

**Figure 2:**
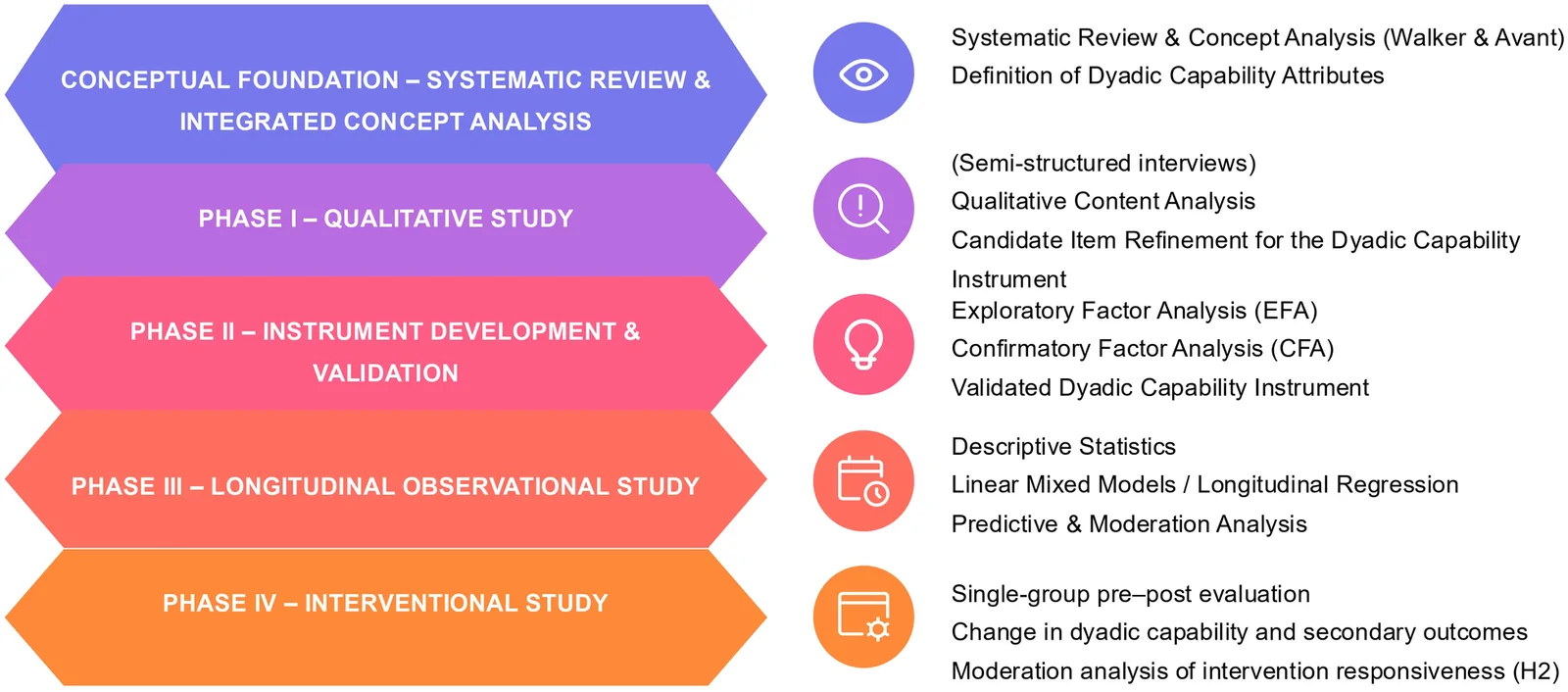
Data analysis plan and qualitative–quantitative integration. The figure illustrates the sequential and integrated data analysis strategy of the study. The conceptual foundation, based on a previously published systematic review and integrated concept analysis, defines the core attributes and conceptual domains of dyadic capability. In Phase I, qualitative data are collected through semi-structured interviews and analysed using qualitative content analysis to inform refinement of the candidate item pool. In Phase II, the instrument undergoes psychometric validation through exploratory and confirmatory factor analyses and reliability testing. In Phase III, longitudinal quantitative analyses using descriptive statistics and mixed-effects models test the predictive and moderating role of dyadic capability over time. In Phase IV, intervention-related changes are evaluated through a single-group pre–post analysis. Finally, qualitative and quantitative findings are integrated through triangulation and meta-inference to further refine the dyadic capability framework. Figure 2 was created by the authors and includes a graphic element from Canva, used under the Canva Content License Agreement.

**Figure 3:**
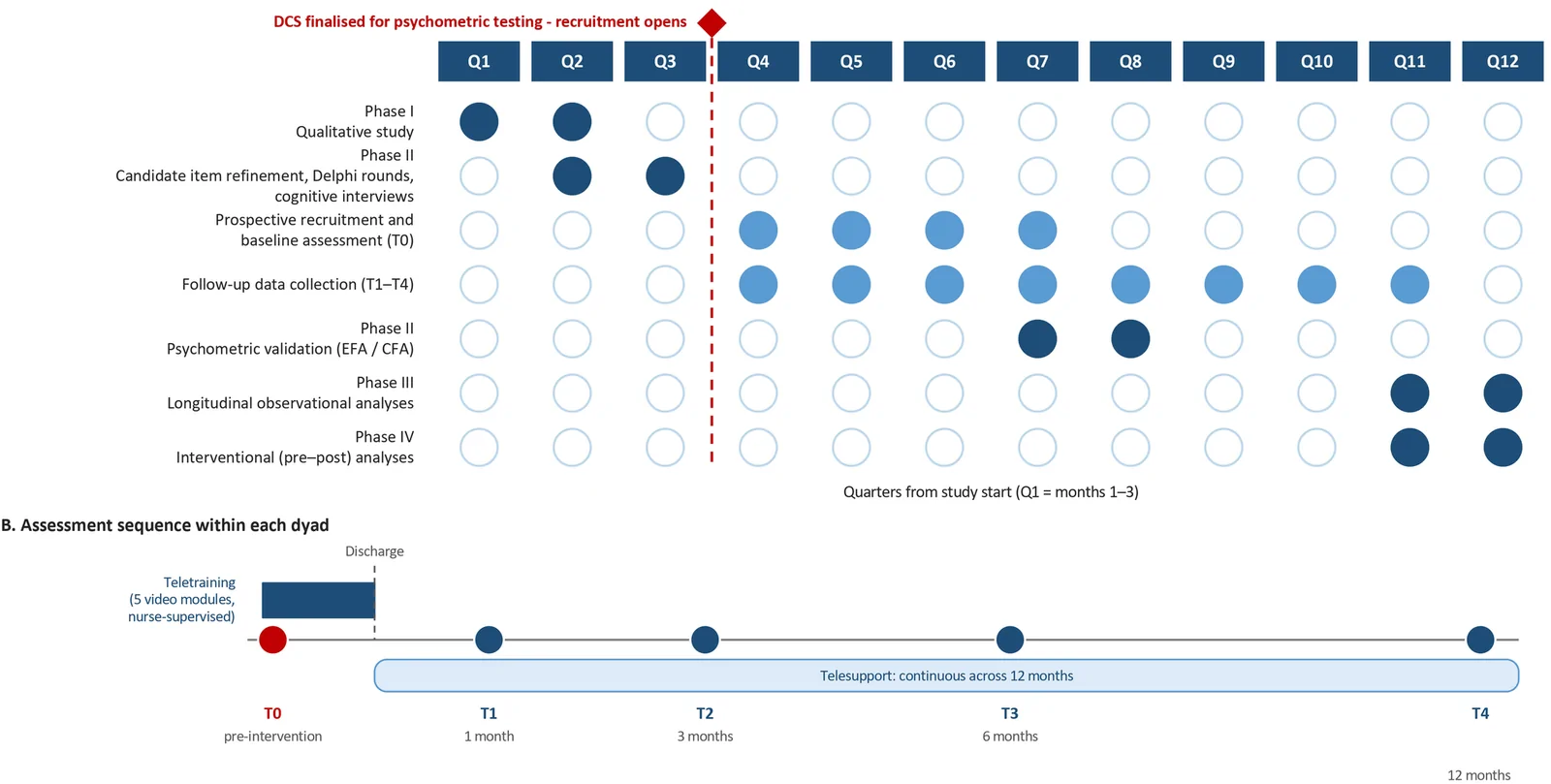
Timeline of the study phases and dyad-level assessment sequence. **(A)** Prospective recruitment opens only after finalisation of the Dyadic Capability Scale (DCS). **(B)** Within each dyad, the baseline assessment (T0) is completed during hospitalisation, before the supervised video-training session and before the first nurse-navigator contact; T1–T4 therefore reflect cumulative exposure to the programme.

### 4.2 Transparency and registration

The study protocol and related materials have been preregistered and are publicly available on the Open Science Framework (https://osf.io/sjdzp).

### 4.3 Participants and setting

A convenience sample of approximately 200 stroke survivor–caregiver dyads will be recruited prospectively from healthcare institutions delivering the structured teletraining and telesupport programme. Dyads will be identified during hospitalisation and enrolled before the first programme session.

Inclusion criteria: a) Stroke survivors aged 18 years or older who are enrolled in the structured teletraining and telesupport programme, with the baseline assessment completed before the first programme session; b) Their informal caregiver, identified as the primary non-professional care provider. Exclusion criteria: a) Severe cognitive or psychiatric impairments that affect the ability to participate. b) Exposure to any component of the teletraining and telesupport programme before completion of the baseline assessment.

Both members of the dyad must be able to provide informed consent and complete self-administered or assisted questionnaires. The caregiver will be identified based on the self-report of the stroke survivor and confirmed by the healthcare staff.

### 4.4 Instrument

#### 4.4.1 Primary outcome

For measurement purposes, dyadic capability is operationalised as the joint freedom and practical opportunity of stroke survivors and their caregivers to convert personal, social, and contextual resources into adaptive caregiving and self-management actions, with self-efficacy, resilience, and motivation hypothesised to influence choice and enactment rather than operate as conversion factors.

The current candidate item pool of the Dyadic Capability Scale (DCS) comprises 57 items derived from the conceptual foundation. This pool will be further refined through the qualitative study, expert review, Delphi consensus, cognitive interviews, and subsequent item-reduction procedures. The candidate items are provisionally organised into four theory-driven domains, self-efficacy, resilience, motivation, and enactment, each expressed across three relational levels: survivor, caregiver, and couple. In the current candidate structure, self-efficacy, resilience, and motivation comprise 35 self-report items intended to be completed independently by each dyad member using role-specific parallel versions, whereas the enactment domain comprises 22 candidate items intended to be rated by a trained clinical nurse assessor who is not involved in the delivery of the D-STEPS programme, on the basis of competence demonstrated during structured assessments. Candidate items are currently rated on a five-point Likert scale (0 = not at all to 4 = extremely). Negatively worded items will be reverse scored as appropriate in the final instrument following item refinement and validation. The scoring structure will be finalized after item refinement and psychometric evaluation. In the current candidate structure, self-efficacy, resilience, and motivation are intended to contribute to a composite index of capability-related psychological domains, whereas the enactment domain is intended to be scored separately. Two dyad-level indices are provisionally proposed: the Dyadic Discrepancy Index, reflecting within-dyad divergence between survivor and caregiver scores, and the Enactment Gap Index, reflecting the discrepancy between self-reported capability-related psychological domains and nurse-observed enactment. Their final operationalization will be refined following the qualitative phase and psychometric evaluation. The dyad is the primary unit of analysis for dyad-level scores and indices. Content validity will be established through expert review, Delphi rounds, and cognitive interviews; psychometric validation of the self-report component will include exploratory and confirmatory factor analyses on independent subsamples, internal consistency (Cronbach's *α* and McDonald's *ω* ≥ 0.70), test–retest reliability, and construct validity through *a priori* convergent and discriminant hypotheses. The nurse-rated enactment domain will be evaluated separately, including inter-rater reliability (91). Because the resilience and self-efficacy domains of the DCS conceptually neighbour established individual-level instruments used here as predictors (the Walsh Family Resilience Questionnaire and the Revised Caregiving Self-Efficacy Scale), discriminant validity will be explicitly tested. The proposed DCS is intended to operationalise these constructs as components of dyadic capability, assessed at survivor, caregiver, and couple levels and complemented by nurse-observed enactment, rather than as individual traits; we therefore hypothesise moderate, not high, correlations with the corresponding predictor scales (r ≈ 0.30–0.50), and we expect the nurse-rated enactment domain and the Enactment Gap Index to show low correlations with any self-report instrument, as no existing measure captures observed dyadic enactment. The risk of construct overlap is thus addressed *a priori* through directional discriminant hypotheses and bifactor modelling.

#### 4.4.2 Secondary outcomes

Stroke-specific QoL will be assessed with the Stroke Impact Scale 3.0 (SIS 3.0) (92), a 59-item instrument assessing physical, cognitive, and social functioning. Items are scored on a 5-point Likert scale, with standardized subscale scores ranging from 0 to 100; higher scores indicate better functioning. The SIS 3.0 has been validated in the Italian stroke population.

Generic QoL will be assessed with the WHOQOL-BREF (13), a 26-item questionnaire covering physical, psychological, social, and environmental domains. Items are scored on a 5-point Likert scale and transformed to a 0–100 score, with higher values representing better QoL. The WHOQOL-BREF will be administered to both stroke survivors and caregivers and has shown adequate validity in the Italian context.

Functional independence will be assessed with the Barthel Index (BI) (93), a 10-item scale evaluating activities of daily living. Scores range from 0 to 100, with higher scores indicating greater independence. The BI has been validated in stroke populations.

Disability will be assessed with the modified Rankin Scale (mRS) (94), a seven-level scale ranging from 0 (no symptoms) to 6 (death), with scores of 1–5 indicating increasing levels of disability. The mRS is widely used and has strong international reliability and validity.

Psychological wellbeing will be assessed with the Hospital Anxiety and Depression Scale (HADS) (95), a 14-item instrument with two subscales for anxiety and depression (7 items each). Each item is scored on a 4-point Likert scale, with subscale scores ranging from 0 to 21; higher scores indicate more severe symptoms. The HADS has been validated internationally and in Italian populations.

Caregiver burden will be assessed with the Caregiver Burden Inventory (CBI) (96), a 24-item instrument covering time dependence, developmental, physical, social, and emotional burden. Items are rated on a 5-point Likert scale, with total scores ranging from 0 to 96; higher values reflect greater burden. The CBI has been validated in Italian caregivers.

#### 4.4.3 Predictors and covariates

Resilience will be assessed with the Walsh Family Resilience Questionnaire (Walsh-IT) (97), a 31-item instrument adapted and validated in Italian. Items are rated on a 5-point Likert scale (1 = very little; 5 = very much), and scores reflect the strength of nine key family resilience processes grouped into three domains: family belief systems, organizational patterns, and communication/problem-solving.

Spiritual wellbeing will be assessed with the WHOQOL-SRPB (98), a 32-item module of the WHOQOL assessing spirituality, religiousness, and personal beliefs. Items are scored on a 5-point Likert scale, with higher scores reflecting greater spiritual wellbeing.

Mutuality will be assessed with the Mutuality Scale (15, 99), a 15-item tool assessing reciprocity, emotional closeness, and shared values. Items are rated on a 5-point Likert scale, with higher scores indicating stronger mutuality.

Preparedness for caregiving will be assessed with the Preparedness for Caregiving Scale (PCS) (99), an 8-item tool evaluating caregivers' perceived readiness to provide care. Items are scored on a 5-point Likert scale, with higher scores denoting greater preparedness.

Self-efficacy will be assessed with the Revised Caregiving Self-Efficacy Scale (CSES) (100), a 15-item instrument assessing confidence in three domains: obtaining respite, responding to disruptive behaviors, and controlling upsetting thoughts. Items are scored on a 5-point Likert scale, with higher scores reflecting greater caregiving self-efficacy.

Health literacy will be assessed with the HLS-EU-Q16, a 16-item short form of the HLS-EU-Q47. Items are answered on a 4-point Likert scale (“very easy” to “very difficult”), dichotomized into easy (1) and difficult (0), with total scores ranging from 0 to 16. Levels are categorized as inadequate (0–8), problematic (9–12), and adequate (13–16). Validated Italian versions are available.

Sociodemographic and clinical variables will also be collected, including age, sex, marital status, education, employment, income, relationship to the patient, stroke type and severity, comorbidities, caregiving duration, and intensity of care.

Perceived social support will be assessed with the Multidimensional Scale of Perceived Social Support (MSPSS) (101), a 12-item instrument assessing perceived support from family, friends, and significant others. Items are rated on a seven-point Likert scale, with higher scores indicating greater perceived social support. The MSPSS has demonstrated adequate validity and reliability in people with chronic diseases (102).

### 4.5 Data collection

Data will be collected once the candidate Dyadic Capability Scale has been finalized for psychometric testing. The instrument will be tested on dyads consisting of stroke survivors and their informal caregivers who will participate in a structured educational intervention. Assessments will be conducted at five timepoints aligned to the D-STEPS programme: T0 (a single baseline assessment completed during the index hospitalisation and before the dyad receives any component of the programme), T1 (1 month), T2 (3 months), T3 (6 months), and T4 (12 months). Quantitative data will be collected at each timepoint through online surveys, telephone interviews, or in-person sessions, depending on participant preference and logistic feasibility. At T0, the enactment domain will be rated by a trained clinical nurse assessor who is not involved in the delivery of the D-STEPS programme, during a dedicated pre-intervention assessment before the first programme session; the same independent-from-intervention assessment procedure will be used at T1–T4.

Secondary outcomes also include functional status and psychological wellbeing of stroke survivors, measured with standardized instruments. Table 2 provides an overview of the timing and procedures for data collection. To reduce respondent burden and minimise attrition risk, the assessment schedule has been streamlined so that selected predictor measures are administered only at T0 or at T0 and T4, while longitudinal outcome measures and selected time-varying contextual measures are retained across follow-up assessments, as detailed in Table 2.

**Table 2:** Data collection schedule and procedures.

Variables	Instruments	Timepoint	Sample
General characteristics			
Sociodemographic characteristics	Ad hoc questionnaire	T0	S-C
Clinical characteristics	Clinical data; Stroke type and severity; Modified Charlson Comorbidity Index	T0	S
Relationship within dyad	Mutuality Scale	T0	S-C
Primary outcome			
Dyadic capability	Newly developed Dyadic Capability Scale (DCS)	T0, T1, T2, T3, T4	S-C
Secondary outcomes			
Stroke-specific QoL	SIS 3.0	T0-T4	S
Generic QoL	WHOQOL-BREF	T0-T4	S-C
Functional independence	Barthel Index	T0-T4	S
Disability	mRS	T0-T4	S
Psychological well-being	HADS	T0-T4	S-C
Caregiver burden	CBI	T0-T4	C
Predictors			
Resilience	Walsh FRQ	T0	S-C
Spiritual well-being	WHOQOL-SRPB	T0, T4	S-C
Caregiver preparedness	PCS	T0, T4	C
Self-efficacy	CSES	T0, T4	C
Health Literacy	HLS-EU-Q16	T0	S-C
Social Support	MSPSS	T0-T4	S-C

S, stroke survivor; C, caregiver; T0, baseline (a single baseline assessment completed during the index hospitalisation and before the dyad receives any component of the programme); T1, 1 month; T2, 3 months; T3, 6 months; T4, 12 months. T0–T4 indicates assessment at all scheduled timepoints from T0 through T4, whereas commas indicate assessment only at the specified timepoints (e.g., T0, T4).

Instruments: SIS 3.0 = Stroke Impact Scale 3.0; WHOQOL-BREF, World Health Organization Quality of Life-BREF; BI, Barthel Index; mRS, modified Rankin Scale; HADS, Hospital Anxiety and Depression Scale; CBI, Caregiver Burden Inventory; WFRQ, Walsh Family Resilience Questionnaire; WHOQOL-SRPB, World Health Organization Quality of Life-Spirituality, Religion and Personal Beliefs; PCS, Preparedness for Caregiving Scale; CSES, Revised Caregiving Self-Efficacy Scale; HLS-EU-Q16, European Health Literacy Survey Questionnaire, 16-item short form; and MSPSS, Multidimensional Scale of Perceived Social Support.

### 4.6 Outcome measures

Primary outcome: dyadic capability (DCS), assessed at T0–T4 (baseline, 1, 3, 6, and 12 months); secondary outcomes: QoL (SIS 3.0; WHOQOL-BREF), functional independence (Barthel Index), disability (mRS), psychological wellbeing (HADS) and caregiver burden (CBI).

### 4.7 Statistical analysis

Sample size estimation was based on three distinct analytical objectives:

- Instrument validation (EFA/CFA): The current candidate DCS comprises 57 items: a 35-item self-report component and a 22-item nurse-rated enactment component, representing distinct measurement sources; sample size adequacy will therefore be considered separately for the two components. Approximately 400 individual responses will be available from 200 dyads for the self-report component. EFA and CFA will be conducted on independent random subsamples defined at the dyad level while accounting for dyadic clustering. The nurse-rated enactment component will be evaluated separately at the dyad level. Sample size adequacy will be reassessed after item reduction based on the final number of items in each component.
- Primary longitudinal outcome: Longitudinal changes in dyadic capability across the five assessment points will be analysed using linear mixed-effects models. Because no prior empirical estimates are available for the newly developed DCS, no separate effect-size-based sample size calculation is specified for this analysis. The overall recruitment target is determined by the more demanding instrument-validation and confirmatory moderation requirements described below.
- Moderation analysis: The primary confirmatory moderation hypothesis (H2) will be tested within the longitudinal mixed-effects model. Given the absence of reliable prior effect-size estimates for the newly developed DCS and its interaction with mutuality, no separate effect-size-based sample size calculation is specified for this analysis. The planned recruitment target of approximately 200 dyads is intended to support the instrument-validation requirements and the planned longitudinal moderation analyses, while allowing for attrition.

Longitudinal changes over time will be analyzed using linear mixed-effects models (LMMs), with time as a fixed effect and random intercepts for dyads (103). Effect sizes (Cohen's d for repeated measures) and 95% confidence intervals will be reported. Secondary outcomes include stroke-specific QoL (SIS 3.0), generic QoL (WHOQOL-BREF), functional independence (Barthel Index), disability (mRS), psychological wellbeing (HADS), and caregiver burden (CBI). These outcomes will be examined longitudinally using the same LMM framework, adjusting for potential confounders (age, sex, baseline functional and psychological status). One moderation hypothesis is designated *a priori* as confirmatory: mutuality (T0) as moderator of the change in dyadic capability from T0 to T4 (H2), tested as a mutuality×time interaction within the mixed-effects model. All other interactions, including those specified under H5, are exploratory: they will be tested one at a time in separate models, with False Discovery Rate correction applied within this family, and reported as effect sizes with 95% confidence intervals rather than as confirmatory findings.

Given the dyadic nature of the data and the theoretical emphasis on interdependent survivor–caregiver processes, Actor–Partner Interdependence Models (APIM) (104) will be used to explicitly model mutual influences within the dyad. APIM will allow simultaneous estimation of actor effects (the individual's capability on their own outcomes) and partner effects (the individual's capability on the partner's outcomes), thereby adequately capturing dyadic interdependence. Actor and partner effects will be estimated to examine how everyone's resources contribute not only to their own capability, but also to their partner's capability within the dyad.

The psychometric properties of the DCS will be evaluated in parallel. Dimensionality will be explored through Exploratory Factor Analysis and confirmed with Confirmatory Factor Analysis (CFA). Model fit will be assessed using *χ*², Comparative Fit Index (CFI), Tucker–Lewis Index (TLI), Root Mean Square Error of Approximation (RMSEA), and Standardized Root Mean Square Residual (SRMR), with acceptable fit defined as CFI and TLI ≥ 0.90, RMSEA ≤ 0.08, and SRMR ≤ 0.08. Measurement invariance across stroke survivors and caregivers will be tested sequentially at the configural, metric, and scalar levels. Reliability will be assessed with Cronbach's *α* (≥0.70) and test–retest intraclass correlation coefficients.

Construct validity will be examined through correlations between DCS scores and validated measures of resilience, mutuality, preparedness, self-efficacy, spirituality, and health literacy. Small-to-moderate correlations (r = 0.30–0.50) are hypothesized. False Discovery Rate (FDR) correction will be applied to control for multiple testing.

Qualitative data from semi-structured interviews will be analyzed using conventional content analysis (84). Two independent researchers will conduct coding and theme development, with discrepancies resolved by consensus. Trustworthiness will be ensured through triangulation, memo writing, and audit trails (105).

Patterns of missing data will be examined using Little's MCAR test and logistic regression models to explore associations between missingness and observed variables (106). Under the MAR assumption, multiple imputation by chained equations will be performed using 50 imputations (107). The imputation model will include variables included in the primary analyses, partner scores, and auxiliary variables associated with missingness. Estimates will be pooled using Rubin's rules (108). In the event of suspected Missing Not At Random (MNAR) mechanisms, sensitivity analyses using pattern-mixture models will be conducted (109).

Finally, mixed-methods integration will be performed through triangulation at the interpretation stage to identify convergence, complementarity, or divergence between quantitative and qualitative results (110), thereby refining the emerging middle-range theory and informing future intervention design.

Descriptive analyses will be conducted in SPSS (version 25); factor analyses, invariance testing, and APIM in Mplus (version 8.9); mixed-effects models and imputation in R. Anonymized data and analysis scripts will be made available via the Open Science Framework https://osf.io/sjdzp.

### 4.8 Dissemination and ethics

This study was evaluated by the Asl Campania Sud Ethics Committee and was approved on 03/05/2023, protocol register No.008787. The study will follow the Declaration of Helsinki (111). Written informed consent will be collected from all participants, including permission to use de-identified data for secondary analyses. Participants may withdraw at any time without consequences. No adverse events are expected, as the intervention is educational and data collection is limited to questionnaires and interviews; any unintended effect, including emotional distress, will be documented, reported to the Ethics Committee where required, and managed through referral to clinical or psychological services. Data will be anonymized, stored on password-protected servers, and accessible only to the research team. No financial compensation will be provided, but participants may request feedback reports.

## 5 Anticipated theoretical contributions

This study advances the understanding of capability as a dynamic context-sensitive construct within stroke survivor–caregiver dyads. Grounded in Sen's Capability Approach (25), it shifts the focus from the mere availability of resources to the real freedom to utilize them to lead a meaningful life. As Haisma et al. (112) highlight, this requires attention to context, predictors, and social relationships that shape caregiving.

Bandura's theory of self-efficacy (113) further highlights that individuals engage more when they feel competent. Fostering capabilities, rather than merely transmitting information, can improve dyadic participation in long-term care and positively affect QoL and rehabilitation adherence.

The published systematic review and integrated concept analysis (46) identified key antecedents, attributes, and outcomes of dyadic capability, including social support, information/resources, relationship quality, and psychological conditions. Emerging evidence (114) reinforces the need to go beyond preparedness, typically defined as perceived readiness, toward a more comprehensive and context-sensitive assessment of real functional adaptation. Educational opportunities and social support appear as critical enablers, transforming knowledge into resources and fostering autonomy (47, 115).

### 5.1 Empirically expected results

Previous studies (33) have demonstrated that caregiver preparedness moderates the impact of patient depression on QoL. Building on this, we propose that capability may represent a more comprehensive concept for understanding the management of post-stroke disability.

Higher dyadic capability scores are hypothesized to be associated with improved psychological wellbeing, reduced caregiver burden, and greater continuity of post-discharge care. These assumptions will be tested through psychometric validation and longitudinal analysis, positioning capability as a more robust and predictive construct than existing measures of caregiver preparedness.

### 5.2 Strengths and limitations

This study introduces a conceptual framework to operationalize capability in stroke care. The longitudinal mixed-methods design captures temporal dynamics and combines subjective and objective perspectives.

During a longitudinal study, attrition may introduce bias and compromise the completeness of the data. To mitigate attrition, multiple contact modalities and reminders will be used; missing data will be handled through multiple imputation under the MAR assumption, with pattern-mixture models as sensitivity analyses. Differential attrition by capability level may restrict the observed range and underestimate associations. Generalizability is limited by convenience sampling, the exclusion of individuals with severe cognitive impairment, and potential cultural influences, and should be tested in other contexts. The protocol designates a single moderation test as confirmatory; all further interactions are exploratory and will require confirmation in an independent, adequately powered study.

Although a convenience sampling strategy will be used, we will recruit from different clinical settings and include a variety of demographic characteristics. Beyond the original study population, additional external validation will be needed for the newly developed capability instrument. Another limitation involves the initial development of the measurement instrument, which may lack established convergent validity until further studies are conducted. The DCS requires cross-validation in independent samples, testing across stroke subtypes, and the definition of a clinically meaningful cutoff. Due to the observational design, causality cannot be established, and reverse causation and unmeasured confounding cannot be excluded. Because the telesupport component is delivered continuously across the follow-up period, changes observed at T1–T4 cannot be attributed to the teletraining component alone; in the absence of a control group, the two components are not separable.

## 6 Conclusions

This study protocol spans from concept clarification to empirical research and culminates in the evaluation of dyadic capability within a real-world teletraining and telesupport context. Studying a multidimensional construct such as capability is methodologically challenging, requiring both psychometric soundness and contextual validity. This study will develop and validate an instrument to assess dyadic capability in stroke survivor–caregiver dyads. Future studies will be required to determine whether dyadic capability can be modified through targeted interventions.

Strengthening capability is expected to foster empowerment and autonomy, in line with person-centered care. This conceptual shift from preparedness to capability reflects a necessary evolution in post-stroke care toward relational and participatory models of health.

From a clinical perspective, this protocol may support rehabilitation nurses and healthcare professionals in actively involving dyads in discharge planning, shared decision-making, and post-acute support, with a focus on co-building adaptive capability.

### 6.1 Future directions

Long-term interventions should go beyond physical rehabilitation and support adaptive dyadic functioning in daily life. This protocol may serve as a methodological foundation for future longitudinal and cross-cultural studies aimed at refining capability as a construct and exploring its integration into health policy. Ultimately, integrating capability-based assessments into rehabilitation pathways can support more equitable, person-centered, and sustainable stroke care models.

## 7 Study status

Study status: Recruitment has not yet commenced. Recruitment is expected to begin in November 2026 and to be completed by November 2027. The final 12-month follow-up is expected to be completed by November 2028. The ethics approval remains valid for the planned study period.

## 8 Patient and public involvement

Stroke survivors and family caregivers will be involved in reviewing and refining the candidate DCS item pool, as described in the Methods section.
